# Can Progressive Supranuclear Palsy Be Accurately Identified via MRI with the Use of Visual Rating Scales and Signs?

**DOI:** 10.3390/biomedicines13051009

**Published:** 2025-04-22

**Authors:** George Anyfantakis, Stamo Manouvelou, Vasilios Koutoulidis, Georgios Velonakis, Nikolaos Scarmeas, Sokratis G. Papageorgiou

**Affiliations:** 1 1st Department of Neurology, Medical School, Eginition Hospital, National and Kapodistrian University of Athens, 72-74 Vasilissis Sofias, 11528 Athens, Greece; smanouvelou@yahoo.gr (S.M.); nscarmeas@med.uoa.gr (N.S.); sokpapa@med.uoa.gr (S.G.P.); 2 1st Department of Radiology, Aretaieion University Hospital, University of Athens Medical School, Vassilissis Sofias 76, 11528 Athens, Greece; 3 2nd Department of Radiology, Attikon University Hospital, Medical School, National and Kapodistrian University, 12462 Athens, Greece; giorvelonakis@gmail.com

**Keywords:** magnetic resonance imaging, parkinsonian syndromes, hot cross bun sign, morning glory sign, hummingbird sign, midbrain atrophy, atrophy of the third ventricle, visual semi-quantitative rating scales

## Abstract

**Introduction:** Neurodegenerative diseases like progressive supranuclear palsy (PSP) present challenges concerning their diagnosis. Neuroimaging using magnetic resonance (MRI) may add diagnostic value. However, modern techniques such as volumetric assessment using Voxel-Based Morphometry (VBM), although proven to be more accurate and superior compared to MRI, have not gained popularity among scientists in the investigation of neurological disorders due to their higher cost and time-consuming applications. Conventional brain MRI methods may present a quick, practical, and easy-to-use imaging rating tool for the differential diagnosis of PSP. The purpose of this study is to evaluate a string of existing visual MRI rating scales and signs regarding their impact for the diagnosis of PSP. **Materials and Methods:** The population study consisted of 30 patients suffering from PSP and 72 healthy controls. Each study participant underwent a brain MRI, which was subsequently examined by two independent researchers in a double-blinded fashion. Fifteen visual rating scales and signs were evaluated, including pontine atrophy, cerebellar atrophy, midbrain atrophy, aqueduct of Sylvius enlargement, cerebellar peduncle hyperintensities, enlargement of the fourth ventricle (100% sensitivity and 71% specificity) and left temporal lobe atrophy (97% sensitivity and 78% specificity). **Conclusions:** Enlargement of the Sylvius aqueduct, enlargement of the fourth ventricle and atrophy of both temporal lobes together with the presence of morning glory and hummingbird signs can be easily and quickly distinguished and identified by an experienced radiologist without involving any complex analysis, making them useful tools for PSP diagnosis. MRI visual scale measurements could be added to the diagnostic criteria of PSP and may serve as an alternative to highly technical and more sophisticated quantification methods.

## 1. Introduction

Progressive supranuclear palsy (PSP) is a gradually progressive neurodegenerative disease characterized by the abnormal accumulation of tau protein in the basal ganglia, midbrain, frontal lobes, subthalamic nucleus and cerebellum. Tau is a microtubule-associated protein that plays a crucial role in maintaining the structural integrity of neurons. In PSP, tau undergoes abnormal modifications, leading to the formation of neurofibrillary tangles (NFTs) and other tau-related pathologies. The above-mentioned modifications disrupt normal cellular functions and result in axonal transport impairment, synaptic dysfunction and eventual cell death [1]. According to the affected brain region, PSP patients may suffer from prominent postural instability, bradykinesia and rigidity, vertical gaze palsy, oculomotor dysfunction, dysarthria and cognitive decline [2]. PSP usually affects older adults and is sub-divided into PSP-Parkinsonism (PSP-P) and PSP-Richardson’s syndrome (PSP-RS), among several other clinical subtypes [3]. The phenotypic spectrum typically encountered in clinical practice is very variable, overlapping with other neurodegenerative disorders, making an accurate diagnosis of PSP challenging [4].

Different sets of diagnostic criteria are available for PSP, with the most accepted ones defined in 2017 through the National Institute of Neurological Disorders and Stroke and Society for PSP (NINDS-SPSP criteria) [5]. According to the NINDS-SPSP criteria, diagnosis is provided in three levels of certainty (possible, probable and certain), with definite PSP provided after a complete examination and confirmation of the clinical data by specific neuropathological findings. Clinically “probable PSP” is defined as patients with vertical supranuclear palsy with early onset of postural instability, whereas “possible PSP” includes patients with early postural instability with saccadic abnormalities or supranuclear gaze palsy [2]. Based on post-mortem evidence, NINDS-SPSP criteria show excellent accuracy for probable PSP and especially for PSP-RS [5,6]. However, their sensitivity is limited in the early stages of the manifestation of the disease, with an average accuracy of 24% [7]. Most patients receive a diagnosis only after the characteristic clinical features (loss of balance with falls and vertical supranuclear palsy) become particularly evident (3–4 years after the onset), while there is also limited sensitivity for PSP syndromes other than PSP-RS [5]. Additional criteria include the Natural History in Parkinson’s Plus Syndromes (NNIPPS) criteria, established in 2009 and supporting only one level of cognitive accuracy [8], as well as the MDS-PSP criteria (Movement Disorder Society Criteria) aiming to optimize the early, correct and specific clinical diagnosis of the disease, also covering PSP phenotypes other than only PSP-RS [5].

Diagnosing PSP can be challenging due to the overlapping symptoms with other neurodegenerative disorders, such as Parkinson’s disease. As clinical signs may be confusing, the use of imaging studies, such as magnetic resonance imaging (MRI), and the interpretation of its findings is of paramount importance for the differential diagnosis of PSP [1,4]. While PSP diagnosis is mainly based on clinical criteria, the visual imaging parameters of brain MRI can serve either as primary or as complementary evidence for the diagnosis. The most frequent MRI findings consist of midbrain atrophy, dilatation of the third ventricle, atrophy of the cerebellum and generalized supratentorial atrophy often with a frontal predominance [9]. Midbrain atrophy in brain MRI is depicted with the hummingbird sign, the Mickey Mouse sign or the morning glory sign [10,11]. Apart from these descriptive MRI features, further clinically useful indexes include the pons/midbrain ratio (ratio of the midbrain surface to the pons surface) [12], the concept of MRPI (Magnetic Resonance Parkinsonism Index), which includes the ranges of the superior (SCP) and middle (MCP) cerebellar peduncles and the surfaces of the pons and midbrain [13], and the distance of the interscapular cistern from the center of the aqueduct at the level of the nipples, corrected for the anterior–posterior ligament distance (MBTegm, midbrain tegmen diameter) [14]. Furthermore, the diagnosis of patients with PSP can be assisted by elements in the signal densities in the different sequences used [15,16]. While these findings are not exclusive to PSP, they contribute to the overall clinical assessment.

High-resolution structural MRI with volumetric analysis allows for the measurement of brain volumes, including atrophy in specific regions associated with PSP, such as the midbrain, frontal cortex and other structures [17]. Voxel-Based Morphometry (VBM) is a neuroimaging technique based on the analysis of the spatial distribution of gray matter and is used in structural MRI to analyze differences in brain anatomy and detect changes in regional brain volume. In PSP, VBM can effectively quantify midbrain atrophy with a greater diagnostic accuracy in comparison to conventional MRI. However, due to being a high-cost and time-consuming application, VBM has not gained popularity in the scientific community [18,19].

We assume that conventional brain MRI methods may present a quick, practical, and easy-to-use imaging rating tool for the differential diagnosis of PSP compared to more sophisticated analytical imaging techniques. The aim of this study is the assessment of the impact of a string of existing visual MRI rating scales and signs for the clinical diagnosis of PSP by studying patients already diagnosed with this neurodegenerative disease.

## 2. Materials and Methods

After approval by the local institutional review board and ethics committee for the purposes of this study, written informed consent was obtained from all patients or their primary caregivers. The population study consisted of 30 patients suffering from PSP and 72 healthy controls. Data was collected withing a period of 5 years among patients visiting the hospital’s neurology service. Patient clinical assessments and diagnoses were performed in the Cognitive Disorder/Dementia Unit Clinic of “Eginition” University Hospital (1st University Department of Neurology) by a single behavioral neurologist, with specific expertise in dementia and movement disorders (S.G.P), using international diagnostic criteria [3]. The control group was recruited from the ongoing ALBION study. They were aged 40 years or older, were evaluated at the cognitive disorders’ outpatient clinic of the Aiginition hospital. All control participants underwent an extensively standardized clinical neurological and neuropsychological assessment. Control participants had been deemed not to suffer from any major neurological disease. One of the healthy control participants had a history of neurological diseases, and all of them had normal neurologic examinations. The disease duration and mini-mental state examination (MMSE) score were also recorded for each participant.

Each study participant underwent a brain MRI. The MRI exams included in the study were performed in the 1st Department of Radiology of the University of Athens Medical School in the Axretaieion University Hospital, with the use of a 1.5 T MRI scanner (Koninklijke Philips N.V., Amsterdam, The Netherlands). MR images include T1-weighted sagittal images and T2-weighted axial and sagittal images; 3D space FLAIR-weighted axial images were also taken. A double-blinded retrospective analysis was conducted by two independent researchers specialized in Radiology and Neuroimaging to estimate the inter-rater agreement. To reduce bias, the radiologists involved in the study did not have access to the diagnosis set by the neurologist, while no imaging data of the patients were disclosed to the neurologist. In the analysis, MRI with technical barriers (incomplete exams, absent sequences, artifacts, etc.) and MRI of patients with other pathologies (e.g., oncology) were excluded.

The following visual rating scales were used, evaluated as an ordinary score from 0 to 3:

(a) According to the NNIPPS MRI rating scale [20], midbrain atrophy, aqueduct of Sylvius enlargement, fourth ventricle enlargement and third ventricle enlargement were assessed.
(b) According to the Comprehensive Visual Rate Scale (CVRS), lateral ventricle enlargement was assessed [21].
(c) According to the Scheltens rating scale [22,23], medial temporal lobe atrophy (left–right) was assessed.

**Figure 1:**
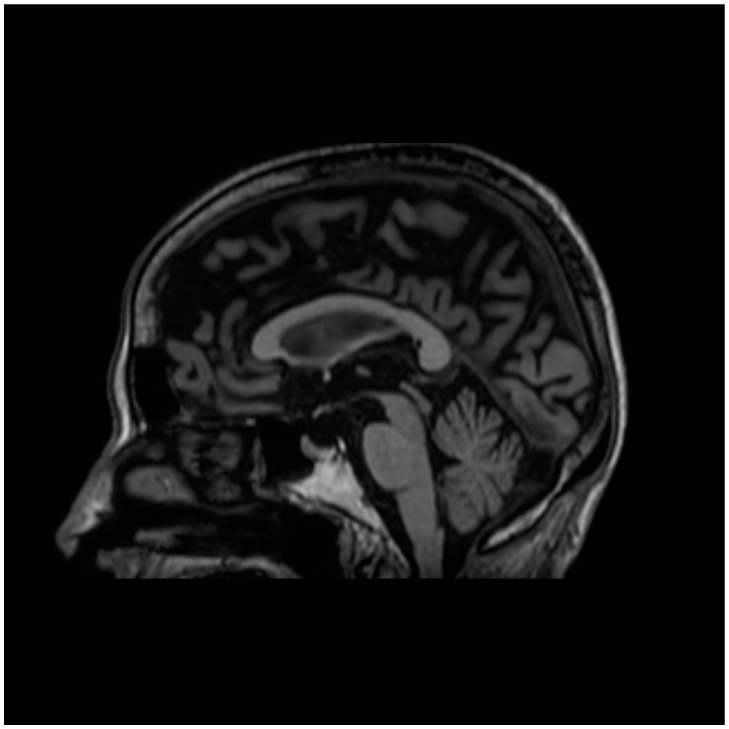
Sagittal MRI T1 scan demonstrating characteristic hummingbird sign in PSP.

**Figure 2:**
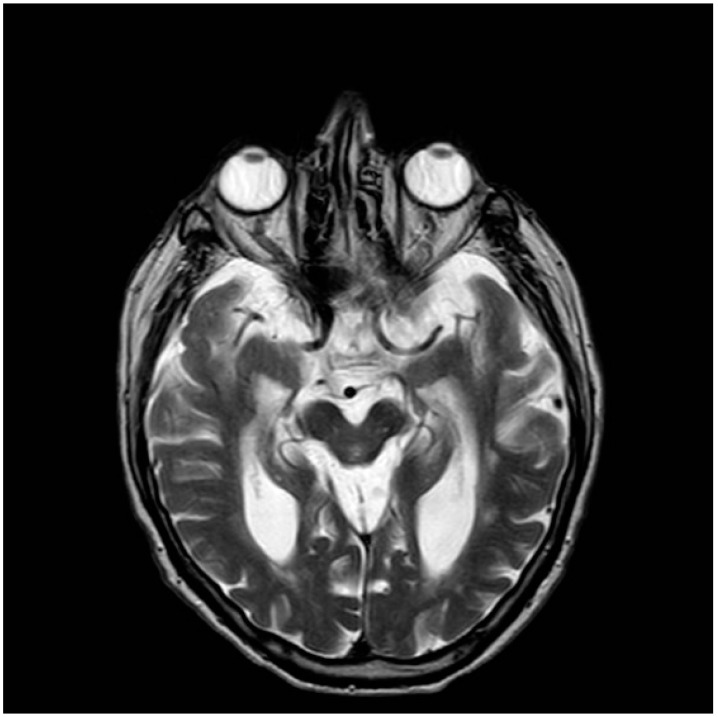
Axial MRI T2 scan demonstrating characteristic morning glory sign in PSP.

The following specific imaging signs were used:

(i) Hot cross bun sign: This refers to atrophy and degeneration observed in the pons. This radiological finding is characterized by a cruciform or cross-like hyperintensity in the pons on T2-weighted or fluid-attenuated inversion recovery (FLAIR) MRI sequences [24].
(ii) Hummingbird sign: In midbrain atrophy, the thinning of the midbrain tegmentum and the widening of the superior cerebellar peduncles contribute to this distinctive imaging pattern. The superior cerebellar peduncles extend upward, and when combined with the atrophy of the midbrain, they create a silhouette reminiscent of a hummingbird (Figure 1) [25].
(iii) Morning glory sign: This refers to the appearance of the tonsils on sagittal MRI images. In individuals with PSP, the cerebellar tonsils often appear elongated and point downward, resembling the shape of a morning glory flower (Figure 2) [25].

### Statistical Analysis

Continuous variables were expressed as the mean ± standard deviation (SD), while categorical variables are expressed as percentages. The normality analysis of the continuous variables was performed using the Kolmogorov–Smirnov test. Inter-observer agreement between the two independent observers was assessed using the linear weighted-kappa values, while the weighted-kappa values’ degree of agreement was defined according to Landis and Koch [25].

The comparison of visual rating scales between PSP patients and the control population was performed using an independent samples *t*-test and Mann–Whitney test. The prognostic ability of visual rating scales to discriminate PSP patients from the control population was examined through a receiver operating curve (ROC) analysis calculating the respective areas under the curve (AUC) with a 95% CI [26]. Furthermore, different cut-off points of these visual rating scales were examined with regard to their sensitivity and specificity. The strongest predictors between scores of the visual rating scales were identified through the logistic regression analysis using the forward LR (Likelihood Ratio) selection method with an ordinal or categorical form for the outcome variable (PSP vs. control). The results of the above analysis are introduced using the Odds Ratio (OR) with a 95% Confidence Interval (CI)

The statistical significance of the results was set at *p* < 0.05, and all executed tests were two-sided. The statistical analysis was carried out using the statistical package SPSS vr21.00 (IBM Corporation, Somers, NY, USA).

## 3. Results

### 3.1. Demographic Characteristics

Brain MRI exams of 30 patients (17 men and 13 women, mean age 69.16 ± 7.09 years) diagnosed with PSP were compared to brain MRI of the controls. The control group included 72 controls (30 men and 42 women, mean age 66.06 ± 6.88 years). The demographic characteristics of both the PSP and control groups are presented in Table 1. There has been a homogeneity of the two compared groups with regard to age (*p* = 0.065), gender (*p* = 0.131), education (*p* = 0.290) and dominant hand (*p* = 0.988), without any significant differences.

### 3.2. Inter-Rater Reliability

The ICC values of the ordinal visual scales, being all higher than 0.90, indicate a perfect agreement between the two independent observers. Additionally, it is notable that the three categorical visual scales had a kappa coefficient value of 1, which again indicates a perfect agreement for the reassessment between A and B researchers (Table 2).

### 3.3. Comparison of MRI Ordinal Visual Rating Scales Between Groups (Control vs. PSP)

The inter-group comparison of MRI visual rating scales indicated that the PSP group had a statistically significantly higher value for all indicators (*p* < 0.005) compared to the control group (Table 3).

### 3.4. Comparison of MRI Qualitative Visual Rating Scales Between Groups (Control vs. PSP)

The comparison of MRI qualitative visual rating scales indicated that the positive hot cross bun sign presented a non-statistically significant difference between the control and PSP groups (2.8% vs. 13.3%, *p* = 0.06). On the other hand, the positive morning glory sign presented a statistically significant difference between the control and PSP groups (5.6% vs. 90%, *p* < 0.0005). Finally, the hummingbird sign also presented a statistically significant difference between control and PSP groups (5.6% vs. 90% *p* < 0.0005) (Table 4).

### 3.5. ROC Analysis for the Differentiation Between Control and PSP Groups

With regard to the ROC analysis, we observe that the index with the highest area under the curve value (AUC) was midbrain atrophy (AUC: 0.946), followed by atrophy of the left temporal lobe (AUC: 0.934) and aqueduct of Sylvius enlargement (AUC: 0.933). With regard to the sensitivity of the indicators, the best value was presented by the enlargement of the fourth ventricle (100%), followed by the enlargement of the third ventricle (97%), midbrain atrophy (97%) and temporal lobe atrophy, both left and right (97%) (Table 5)

**Figure 3:**
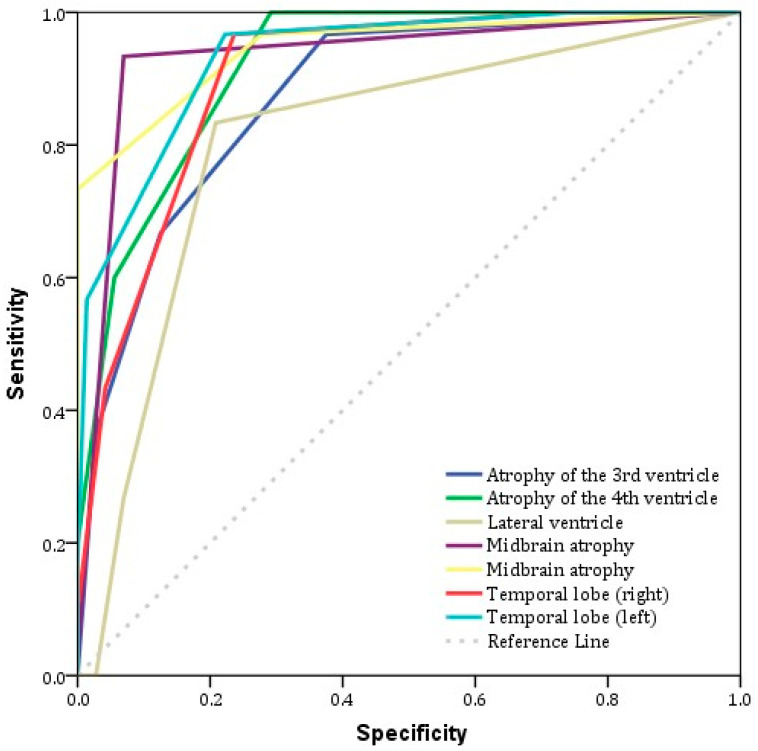
ROC analysis of MRI visual rating scales for differentiation between control population and PSP group.

The highest value for specificity was presented by aqueduct of Sylvius enlargement (93%) (Table 5). With regard to the optimum combination of sensitivity and specificity, the indexes with the best predictive value for the identification of PSP patients as opposed to the normal population were aqueduct of Sylvius enlargement (93% sensitivity and 93% specificity), followed by the enlargement of the fourth ventricle (100% sensitivity and 71% specificity) and left temporal lobe atrophy (97% sensitivity and 78% specificity) (Table 5) (Figure 3).

### 3.6. Logistic Regression Using the Forward LR Method for the Examination of the Predictive Ability of MRI Visual Rating Scales for Differentiating PSP Patients from the Control Population

According to logistic regression analysis, the strongest predictors for differentiation between the control and PSP groups were the morning glory sign and aqueduct of Sylvius enlargement. A positive result for the morning glory sign was related to PSP diagnosis compared to the control [OR (95% CI): 36.1 (5.3–246.5), *p* < 0.005]. Values of aqueduct of Sylvius enlargement above 0.5 were related to PSP diagnosis compared to the control [OR (95% CI): 48.3 (6.7–346.8), *p* < 0.005] (Table 6).

## 4. Discussion

The results of the present study have shown that visual MRI rating scales and signs have a high sensitivity and specificity in the diagnosis of PSP that is comparable with modern computational methods of quantification, like VBM, which are usually more expensive and time consuming. Among the 15 visual scales evaluated, 3 have been found to provide satisfying results in terms of sensitivity and specificity for PSP: aqueduct of Sylvius enlargement (93% sensitivity and 93% specificity), left temporal lobe atrophy (97% sensitivity and 78% specificity) and right temporal lobe atrophy (97% sensitivity and 76% specificity). Significant atrophy in the temporal lobe, particularly the left side, is not a classical feature of PSP but may occur in cases of PSP with predominant behavioral or cognitive symptoms, sometimes referred to as the PSP-cognitive or PSP-behavioral variant [1]. The above-mentioned results of our study may also be attributed to our study group demographics.

Although conventional MRI has improved diagnostic accuracy for PSP, its characteristic imaging findings may not always be present. Furthermore, the interpretation of conventional MR images remains subjective [27]. Recently, advanced neuroimaging methods, such as structural MRI, VBM and diffusion tensor imaging (DTI), have increasingly been used for the differential diagnosis of PSP, as they can objectively quantify the atrophic changes of gray matter and white matter and allow for clinico-radiological correlation [19]. By analyzing structural differences in the brain, VBM allows for the identification of patterns of atrophy or changes in the regional brain volume associated with PSP. VBM can quantify midbrain atrophy and frontal cortex involvement, helping to differentiate PSP from other neurodegenerative disorders with overlapping symptoms. VBM depicts PSP-specific gray matter atrophy in the thalamus, basal ganglia, midbrain, insular cortex and frontal cortex [28]. Additionally, PSP patients present enlargement in the ventricular system (*p* < 0.001), involving the lateral, third and fourth ventricles. According to Price et al., VBM-depicted midbrain atrophy achieved a 83% sensitivity and 79% specificity in PSP diagnosis [19]. The results of the present study regarding midbrain atrophy were comparable (97% sensitivity and 72% specificity) using only visual MRI rating scales. VBM may reveal regional atrophy, but it does not directly identify the underlying molecular pathology, such as the accumulation of tau protein, which is a hallmark of PSP.

Although the hummingbird sign is widely used for the diagnosis of PSP, it is a subjective marker, and there is no recognized consensus on cut-off values or mandatory features. Moreover, there is a plethora of different species of hummingbirds in nature, making its use unreliable [29]. However, the hummingbird sign and morning glory sign are widely used in the literature. In a study by Massey et al., conventional structural MRI had a higher specificity but lower sensitivity compared to the clinical diagnosis in PSP. The hummingbird sign and morning glory sign both had 100% specificity for PSP but lower sensitivities (68.4% and 50%, respectively) [30]. In a recent study by Zhao et al., enlargement of the third ventricle had a 79% sensitivity in PSP diagnosis, while the sensitivity of the hummingbird sign was only 58% [31]. A recent study by Wattjes et al. found that the hummingbird sign had a 58% sensitivity and 81.5% specificity, while the morning glory sign had a 36% sensitivity and 93% specificity for PSP diagnosis. The authors concluded that fully automatic classification of volumetric MRI measures using machine learning methods outperforms visual MRI analysis without and with planimetry or volumetry support [32]. In the present study, the sensitivity of both the hummingbird sign and the morning glory sign was 90%, and their specificity reached 94% for the diagnosis of PSP, suggesting that simple visual MRI rating may be sufficient for the diagnosis of PSP.

Midbrain atrophy and superior cerebellar peduncle atrophy may be used in the differential diagnosis of PSP-RS from other parkinsonian syndromes [30]. These findings can be tracked longitudinally using diffusion tensor imaging (DTI) [33]. Another cohort study by Morelli et al., including pathologically confirmed PSP cases, observed that the MRPI had a 100% sensitivity and 99–100% specificity for the diagnosis of PSP-RS [34]. The pons/midbrain ratio, with a 0.52 cut-off value, had a 100% specificity for the diagnosis of pathologically confirmed PSP [12]. Linear MRI measures have been omitted in the present study given the fact that these measurements usually demand more time. The present study aims to provide a quick and practical visual tool for the diagnosis of PSP.

The results of the present study have shown that a relatively skilled neuroradiologist can use a small number of simple MRI findings and reach a conclusion within a minimum timeframe, which is by far faster than the assessments required using more sophisticated methods. Furthermore, amongst the above-mentioned visual tools, a general radiologist might select to use only three of them. As suggested by the detailed statistical analysis (ROC analysis) in the present research, the most important tools for the differential diagnosis between PSP consist of the morning glory sign (sensitivity 90% and specificity 77%) and midbrain atrophy combined with atrophy of the third ventricle (AUC 0.840). Accordingly, the morning glory sign, as well as the combination of midbrain atrophy and third ventricle enlargement patterns, may be used exclusively as an even faster visual tool for the differentiation of these two entities, allowing the radiologist to complete the ratings within 1–2 min.

We believe that a conventional MRI assessment is inferior to advanced MR imaging for the identification of neural tissue atrophy, but these advanced techniques are not practical for daily use, as they are time consuming and require advanced analysis methods. When PSP diagnosis is based on histological findings, radiological diagnosis based on conventional MRI has a much higher specificity in comparison to clinical diagnosis [30]. Therefore, the identification of proper findings of conventional MRI with great diagnostic value for PSP is of paramount importance. The most important role of conventional MRI in the diagnosis of PSP is the detection of a variety of PSP-related abnormal findings, especially atrophic changes. For the evaluation of delicate nervous structures, such as the midbrain and superior cerebellar peduncles, the use of a combination of axial, sagittal and coronal directions is adequate [17]. Midbrain atrophy, aqueduct of Sylvius enlargement and temporal lobe atrophy combined with the morning glory sign and hummingbird sign can provide a simple, easy to use and fast rating tool for the differential diagnosis of PSP.

The present study contains certain limitations. Apart from the retrospective design of the study, the sample size was somewhat small. Moreover, the reported sensitivity and specificity rates in our study were based on PSP clinical criteria and not on histological diagnosis. Thus, the possibility of misdiagnosis cannot be excluded. Finally, the duration of the disease was not reported. More groups of patients suffering from PSP variants would be a useful addition to the study so that multiple comparisons could be performed.

## 5. Conclusions

The present study has concluded that the use of visual ratings of conventional brain MRI is a fast, cheap and effective option for the diagnosis of PSP, with a relatively high sensitivity and specificity. Midbrain atrophy, aqueduct of Sylvius enlargement and temporal lobe atrophy combined with the morning glory sign and hummingbird sign can provide a simple, easy to use and fast rating tool for the differential diagnosis of PSP. MRI visual scale measurements could be added to the diagnostic criteria of PSP and may serve as an alternative to highly technical and more sophisticated quantification methods. Taking into consideration the scarcity of systematic studies assessing histologically proven PSP, especially atypical subtypes, with advanced MRI techniques, high-quality studies using these techniques are needed in order to fully elucidate the role of MRI in PSP diagnosis.

## Figures and Tables

**Table 1:** Comparison between groups (control vs. PSP) of demographic characteristics.

Demographic Characteristics	Control (ν = 72)	PSP (ν = 30)	*p*-Value
Age (years), mean ± SD	66.06 ± 6.88	69.16 ± 7.09	0.065
Gender, male/female; n (%)	30 (41.7)/42 (58.3)	17 (56.7)/13 (43.3)	0.131
Εducation (years), mean ± SD	12.92 ± 3.73	12.17 ± 3.00	0.290
Dominant hand, right/left/ambidextrous; n (%)	65 (90.3)/ 5 (7.0)/ 2 (2.8)	27 (90.0)/ 2 (6.7)/ 1 (3.3)	0.988

**Table 2:** Ιnter-observer reliability between the two raters.

	**ICC**	**95% CI**	***p*-Value**
Enlargement of the third ventricle	0.935	0.90–0.96	***<0.005***
Enlargement of the fourth ventricle	0.891	0.83–0.93	***<0.005***
Lateral ventricle enlargement	0.968	0.95–0.98	***<0.005***
Aqueduct of Sylvius enlargement	0.938	0.90–0.96	***<0.005***
Midbrain atrophy	0.966	0.95–0.98	***<0.005***
Atrophy of the right temporal lobe	0.965	0.95–0.98	***<0.005***
Atrophy of the left temporal lobe	0.961	0.94–0.98	***<0.005***
	**Kappa**	**95% CI**	***p*-Value**
Hot cross bun sign	1.00	1.00–1.00	***<0.005***
Morning glory sign	1.00	1.00–1.00	***<0.005***
Hummingbird sign	1.00	1.00–1.00	***<0.005***

**ICC**: Intra-class Correlation Coefficient, **CI:** Confidence Interval.

**Table 3:** Comparison of MRI visual rating scales between groups (control vs. PSP). All variables are presented as the mean ± SD.

	Control (ν = 72)	PSP (ν = 30)	*p*-Value
Enlargement of the third ventricle	0.53 ± 0.79	2.00 ± 0.91	***<0.005***
Enlargement of the fourth ventricle	0.35 ± 0.59	1.80 ± 0.76	***<0.005***
Lateral ventricle enlargement	0.31 ± 0.68	1.10 ± 0.66	***<0.005***
Aqueduct of Sylvius enlargement	0.08 ± 0.33	1.17 ± 0.59	***<0.005***
Midbrain atrophy	0.28 ± 0.45	1.87 ± 0.73	***<0.005***
Atrophy of the right temporal lobe	1.01 ± 0.80	2.50 ± 0.73	***<0.005***
Atrophy of the left temporal lobe	0.99 ± 0.72a	2.70 ± 0.79	***<0.005***

**Table 4:** Comparison of qualitative scales between groups (control vs. PSP). All variables are presented as n (%).

		Control (ν = 72)	PSP (ν = 30)	*p*-Value
Hot cross bun sign	negative	70 (97.2)	26 (86.7)	*0.06*
	positive	2 (2.8)	4 (13.3)	
Morning glory sign	negative	68 (94.4)	3 (10.0)	***<0.005***
	positive	4 (5.6)	27 (90.0)	
Hummingbird sign	negative	68 (94.4)	3 (10.0)	***<0.005***
	positive	4 (5.6)	27 (90.0)	

**Table 5:** ROC analysis (control vs. PSP). **AUC**: area under the curve. **CI:** Confidence Interval.

	AUC	95% CI		*p*-Value	Sensitivity	Specificity
Enlargement of the third ventricle	0.874	0.80	0.95	<0.005	97%	63%
Enlargement of the fourth ventricle	0.919	0.87	0.97	<0.005	100%	71%
Lateral ventricle enlargement	0.808	0.71	0.90	<0.005	83%	79%
Aqueduct of Sylvius enlargement	0.933	0.87	0.99	<0.005	93%	93%
Midbrain atrophy	0.946	0.89	1.00	<0.005	97%	72%
Atrophy of the right temporal lobe	0.903	0.84	0.96	0.013	97%	76%
Atrophy of the left temporal lobe	0.934	0.89	0.98	<0.005	97%	78%

**Table 6:** Logistic regression using the forward LR method for differentiation between the control and PSP groups. **OR**: Odds Ratio, **CI:** Confidence Interval.

	Reference Category	OR	95% CI		*p*-Value
Aqueduct of Sylvius enlargement	below 0.5	48.25	6.71	346.76	***<0.005***
Morning glory sign	negative	36.04	5.27	246.49	***<0.005***

## Data Availability

The data that support the findings of this study are not openly available due to reasons of sensitivity and are available from the corresponding author upon reasonable request. MRI data are located in controlled access data storage in the Cognitive Disorder/Dementia Unit Clinic the Cognitive Disorder/Dementia Unit Clinic of the 1st University Department of Neurology at the University Hospital “Eginiteion”.
